# Exploring the Role of Contextual Factors in Medicaid Nursing Homes' Performance: A Qualitative Perspective

**DOI:** 10.1093/geroni/igab046.075

**Published:** 2021-12-17

**Authors:** Justin Lord, Midge Ray, Amy Landry, Heather Lee, Nataliya Ivankova, Ivan Herbey, Robert Weech-Maldonado

**Affiliations:** 1 Louisiana State University at Shreveport, Bossier City, Louisiana, United States; 2 University of Alabama at Birmingham, Birmingham, Alabama, United States; 3 UAB, Birmingham, Alabama, United States

## Abstract

This study explored the role of tested contextual factors (structural, market, and management) in high Medicaid (under resourced) nursing homes performance. Four nursing homes in geographically diverse states were purposefully selected for site visits based on high and low performance (quality/ profitability) indicators. Eight nursing home administrators and directors of nursing, and twenty-one nursing staff (RNs, LPNs, and CNAs) and providers of support services were interviewed. Data were analyzed using an inductive thematic approach with NVivo 12 Plus. Within and across case analysis was used to compare participants’ perspectives across nursing homes and across administrators and staff. Several themes provide insight into varied influences of contextual factors on these nursing homes’ performance: focus on quality care, team-based approach, community support and engagement, and staffing retention. Providing quality care to residents was strategic priority in all facilities, which was enhanced by an adopted team-based leadership approach, open-door policy and home-like atmosphere. Community reputation and availability of local training opportunities for CNAs affected nursing staffing which some facilities addressed using creative retention strategies. These research findings will facilitate interventions, such as leadership training and organizational development activities, aimed at improving the performance of low performing facilities in terms of lower costs and better quality.

